# Do women in science form more diverse research networks than men? An analysis of Spanish biomedical scientists

**DOI:** 10.1371/journal.pone.0238229

**Published:** 2020-08-27

**Authors:** Adrián A. Díaz-Faes, Paula Otero-Hermida, Müge Ozman, Pablo D’Este

**Affiliations:** 1 INGENIO (CSIC-UPV), Universitat Politècnica de València, Valencia, Spain; 2 Institut Mines-Telecom Business School, Evry, France; Indiana University Bloomington, UNITED STATES

## Abstract

This paper examines the role of gender in the formation of research collaboration networks, by investigating the composition of networks through connections to diverse professional communities. Drawing on an ego network approach, we examine gender differences among researchers’ networks in terms of partner diversity, openness and brokerage roles. We use data from 897 valid responses to a questionnaire administered to biomedical scientists in Spain, which enquired into multiple aspects of personal research networks. Our findings show that women form more diverse networks and brokerage triads than men. This result is reinforced if we consider the most heterogeneous brokerage triads in terms of professional differences among network partners (i.e., consultant and liaison). Our results suggest that women are more likely to access non-redundant knowledge and richer research perspectives via their knowledge-flow intermediary roles. This research suggests the need for analyses of gender and networks that go beyond a gender-to-gender approach.

## Introduction

This paper investigates the gender dimension in scientific research networks, by examining the type of networks formed by men and women and focusing on the professional diversity of network partners. We contribute to two complementary, but largely unconnected literature streams: work on networks, which includes studies investigating brokerage roles, and work on gender in the context of innovation and scientific research, which, among other aspects, examines professional barriers and workplace inequalities.

Drawing on an ego network approach [1], we examine gender differences in researchers’ personal networks in terms of partner diversity, openness [2] and brokerage roles [3, 4]. Brokerage profiles allow us to operationalise the structure and composition of these personal networks and to explore whether gender differences matter for the professional diversity of the network partners involved.

Our analysis of the relation between gender and networks is conducted in the specific context of biomedicine where translational research has become a policy priority, with calls for research involving not only basic and clinical researchers but also medical practitioners, industry partners and patients. This context provides a unique opportunity to examine the relationships between multiple social network configurations [5] and gender. On the one hand, because translational research often requires the formation of highly heterogeneous networks in terms of their composition and on the other, because biomedical research is characterised as more gender-balanced, compared to other scientific environments.

We find that women’s networks are more diverse in terms of partners’ professional affiliations and also that women assume more heterogeneous brokerage positions than men. These results suggest that, in their research activities, women form ties to individuals from a wider range of organisational environments and professional communities. Future work could explore whether our findings are generalisable to other research contexts. Also, viewed through a conceptual lens, our findings suggest that future research should go beyond a gender-to-gender approach and, as we propose in this study, include an analysis of gender differences in intermediation roles involving heterogeneous actors in research networks.

The paper is organised as follows. The first section provides an overview of the gender and networks literature. In the second and third sections, we formulate our hypotheses and present our results. The paper concludes with a discussion of the main theoretical and empirical findings.

## Theoretical background: Gender, science, and networks

### Gender imbalance in science

Women in professional environments face a range of barriers that result in their being highly disadvantaged during their career development, compared to men. For instance, women in senior management positions sometimes are seen as outsiders and lacking legitimacy. This is due to the prevalence of male-dominated workplaces and organisational cultures and a predominance of men in top positions [6–10], which makes it difficult for women to gain support from relevant peers for proposed action-plans [11]. As these studies suggest, a gender imbalance in professional environments results in fewer opportunities for advancement in the organisation and less access to elite networks.

The metaphor of a leaky pipeline has been used in science to describe the barriers faced by women at the different stages in their professional careers, which eventually may cause them to leave academia [10, 12]. Gender disparities are evident as early as graduate education; it has been shown that, compared to men, women have inferior access to research opportunities, social support, equipment and mentoring opportunities [13, 14]. Female job applicants continue to be perceived (especially by elite male faculty) as less competent and less attractive hires for science faculty positions, than their male counterparts [13, 15]. In a recruitment context, much emphasis is put on relationships and family status of women [13, 16], while women in tenure track positions suffer significant disparities in pay rates and barriers to promotion compared to men [16, 17]. In general, women are subject to many more institutional constraints than men [18]. In the field of biomedicine, women account for nearly 60% of life sciences and health doctorates [19], but their presence in senior academic positions is not equivalent (about 36% of assistant professors and 18% of full professors are women) [15].

Although there are gender disparities and difficulties particular to women in research environments, there is a strand of research that suggests that in interdisciplinary research fields, they might be advantaged [20–22], showing that female researchers are more heavily involved in interdisciplinary research [20, 23]. Rhoten and Pfirman [20] offer some possible explanations. First, the alleged differences between masculine and feminine epistemological standpoints. While the literature associates the former to objective rationality, the latter are associated to affectual rationality, holism and the possibility of a multiplicity of truths [20, 24]. Second, research suggests that there might be differences between men and women in terms of the types of activities that attract them and there is ample evidence in psychology showing that women’s idealised jobs involve "people" and are "problem oriented" whereas men’s idealised jobs focus on "things" and fundamental theories [25, 26]. Underlying these differences, are the structural positions of men and women in society, which are reproduced through education systems that associate male students to STEM fields [26, 27]. Third, interdisciplinary fields often lack clear status structures and tend to be composed of loosely connected groups of researchers who may not be bound by the norms and values typical of core disciplinary domains. The dominance of a masculine culture in these core disciplines can cause a repositioning of women scientists towards more peripheral areas in multiple fields [20].

In gender unequal contexts, the research networks established by women scientists could help to counter many gender imbalances through the formation of distinctive network patterns that disrupt routinised practices and create new opportunities for greater social legitimacy among peers [6, 7, 28, 29]. Although the collaboration patterns and networking strategies of women in science have been investigated, little is known, from a gender perspective, about the patterns of collaboration in fields that are both cross-disciplinary and involve multiple professional communities.

### Networking patterns and gender

Network research suggests that male and female networking patterns differ. There is a large body of work in the context of scientific research, which shows that not only is research performance linked closely to networks [30–32] but also collaborations have a strong gender component [8, 33–36]. For example, it is suggested that there are differences in how women and men perceive institutional, economic and other barriers to collaboration (e.g., men express more frustration about external conditions such as lack of resources for collaboration, while women tend to blame their inability to access the resources needed for collaboration) [18, 22]. Also, the effect of collaboration patterns on research performance can be different for men and women, usually to the latter’s disadvantage (e.g., stereotyping of women in male-dominated teams and fewer opportunities to benefit from structural holes) [29, 36, 37]. However, it has been shown, also, that women develop specific collaboration strategies to cope with the problems they experience [22, 36, 38]. Bozeman and Gaughan [34] found that women are involved in a higher number of research collaborations than men, while there is evidence, also, that women’s networks are more egalitarian [39].

Overall, the literature warns against controversial essentialist explanations. It suggests, instead, that these differences derive from men’s and women’s different social structural locations, which tend to shape their networking behaviours, rather than from contrasting dispositions based on gender per se [18, 20, 39, 40].

In what follows, we explore the relationship between gender and scientific networks, taking into consideration four network aspects. First, network composition in terms of professional diversity of partners. Second, degree of network openness, which measures the opportunities to act as an intermediary among different actors. Third, the range of brokerage roles, which includes both network openness and network partner type. Fourth, the dissimilarities among brokered partners, that is, the professional disparity between researcher and partners.

#### Diversity of professional collaborators

Few studies that consider gender explicitly focus on the formation of ties with different types of actors in research and innovation activities [41]. Since social network research highlights the importance of network composition for providing individuals with access to dissimilar, non-overlapping knowledge, examining the relationship between gender and network composition becomes a compelling research avenue.

**Fig 1:**
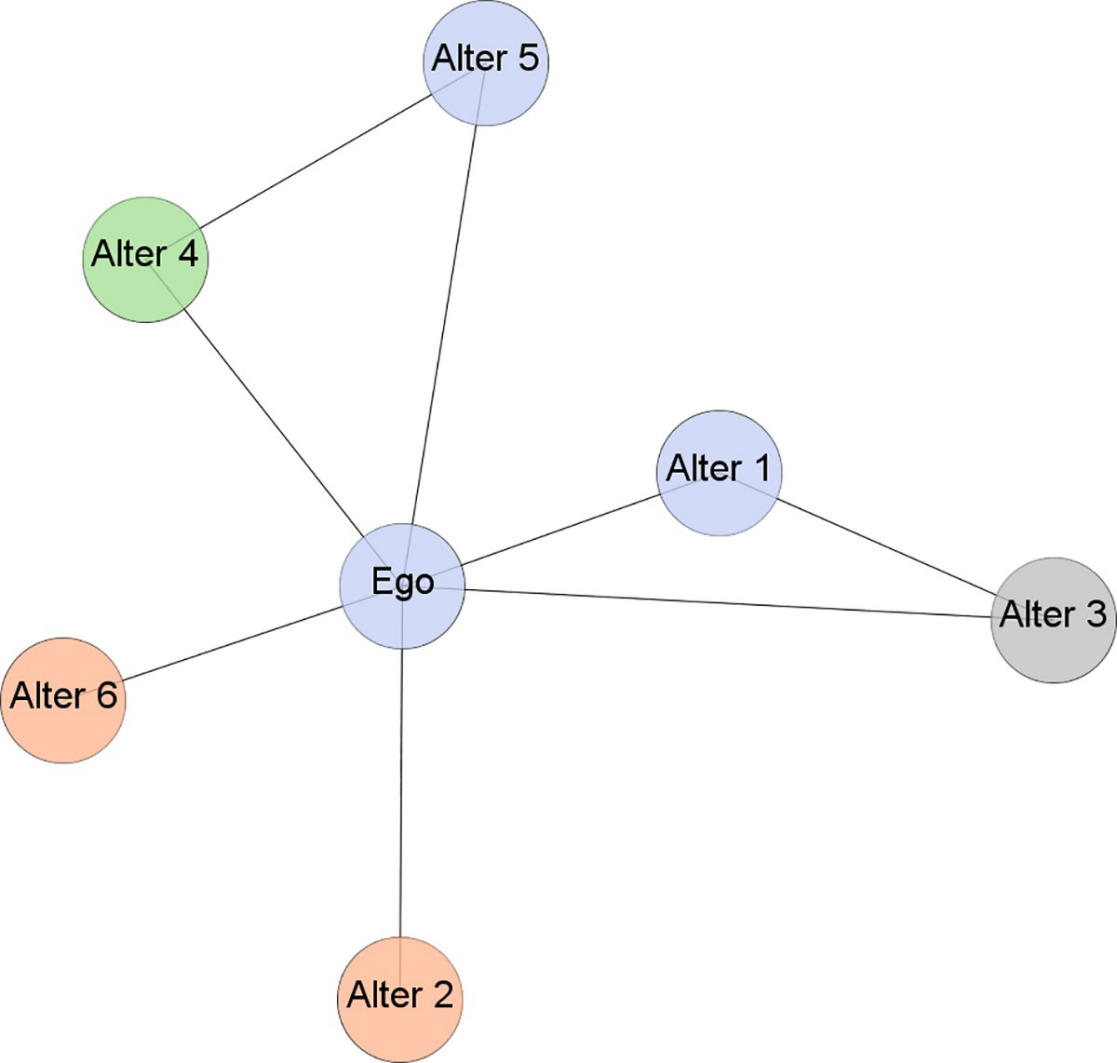
Diverse network composition. The different node colours indicate belonging to different professional communities. Ego networks refer to networks formed around a particular actor—the ego; the nodes to which the ego is directly connected are known as alters.

Ties among dissimilar actors can be understood from the perspectives of demography, ethnicity, occupation or professional community membership (Fig 1 is an example of a diverse ego network). Knowledge related to particular professional communities represents a distinctive individual feature, since actors are influenced by their communities’ organisational practices, norms and values. This defines the importance of establishing ties to individuals in different professional communities [42–44], since they expose the focal actors to alternative ways of thinking that favour knowledge recombination and innovation [45–47].

Diversity in research partner types is important for several reasons. First, there is abundant evidence suggesting the positive effects of network partner diversity on promotion prospects, access to resources and visibility [43, 44, 48, 49]. Second, these positive effects might be especially important for reducing the largely peripheral positioning of women vis a vis the male-dominated research corpus. Research shows that there are differences in the patterns of recognition related to core and peripheral actors [50], and that a more diverse network is especially beneficial for peripheral actors to compensate for their less advantageous structural position compared to core actors [20].

Previous studies on gender and network diversity focus, mostly, on homophily, that is, the extent to which women and men differ in terms of network alter gender [6, 7, 8, 39, 51] (for exceptions to this approach on homophily, see, for instance [37]). Overall, these studies suggest that women tend to build less homophilic networks than men [6, 39]. We extend this line of research by looking at the diversity of women’s alters in terms of professional communities. Since women in science can benefit from the inclusion in their networks of diverse partners, which help to overcome the negative effect of a peripheral position, we hypothesise that:

**Hypothesis 1:** *Women’s research networks are more likely to include a more diverse range of partners*, *in terms of the professional communities to which network partners belong*.

#### Openness of professional networks

**Fig 2:**
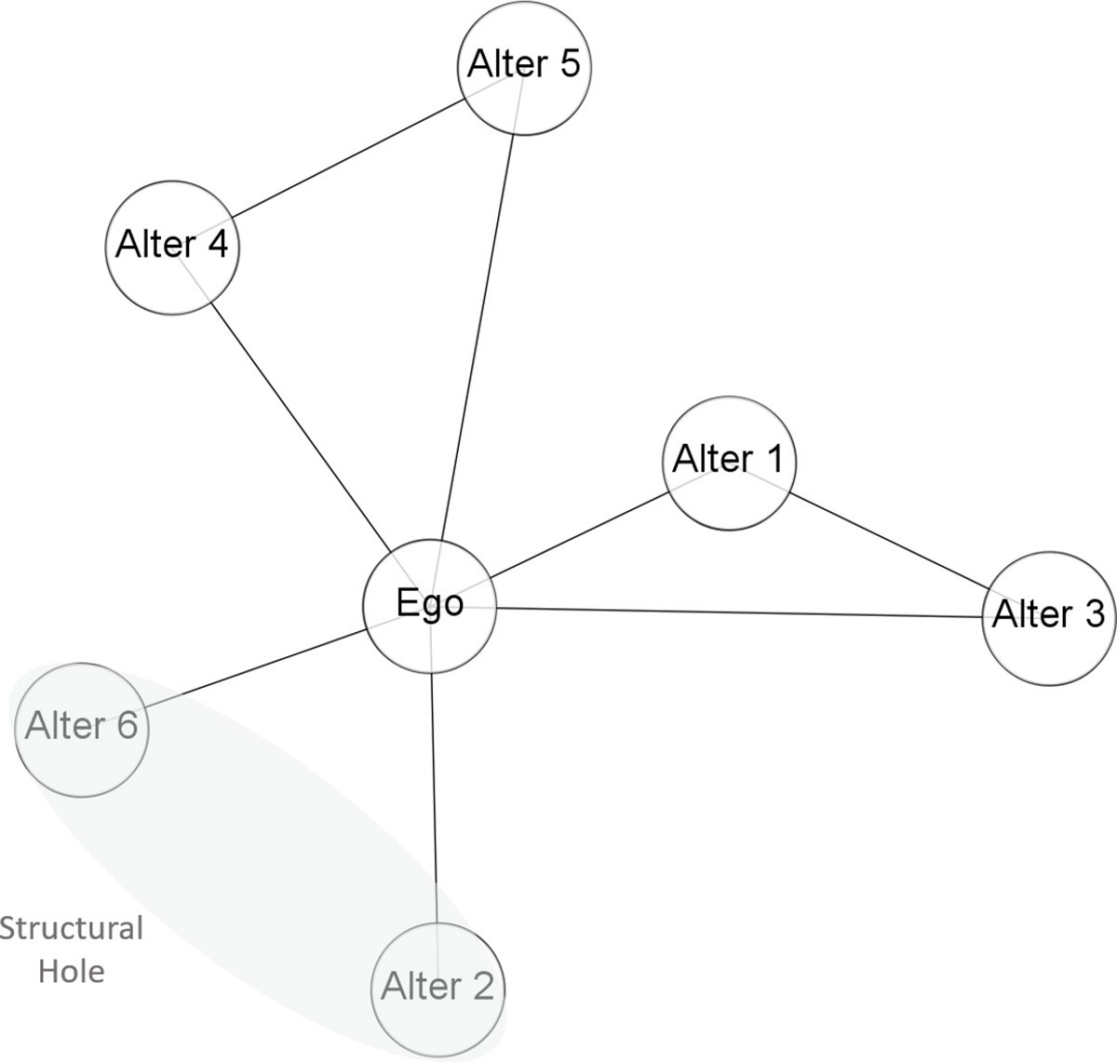
Brokerage based on structural features. Brokerage opportunities are in close connection with structural holes theory [2], which points to the competitive advantage of actors that span or intermediate between otherwise disconnected alters.

Access to relevant knowledge and research effectiveness are also influenced by an alternative mechanism, the degree of openness/closedness of the research network. The network literature considers brokers as critical for reducing the barriers to collaboration and translating research discoveries into practical applications [43, 52]. Brokerage measures the extent to which an actor is capable of linking others who are otherwise not connected to each other. Therefore, brokerage can be described as a “relation involving three actors, two of whom are the actual parties to the transaction and one of whom is the intermediary or broker” [3] (p. 9). Since brokers mediate between otherwise disconnected alters, they achieve privileged access to non-redundant information and are exposed to diverse interests and perspectives [2, 47, 53]. Brokerage opportunities derive from weak network cohesion (i.e., weak connectivity among network partners), and thus, open (as opposed to closed) network structures [2]. Fig 2 depicts a brokerage position within an ego network.

Previous research suggests there are differences in how open and closed networks (few ties between alters vs. highly embedded networks) influence men’s and women’s performance in different contexts [29, 37, 54] and the extent to which gender influences network openness. While some studies suggest that women scientists form more open networks compared to men [55], others show that when building social capital, women establish networks with higher levels of connectivity among partners [56].

As argued in relation to network composition, the research networks established by women scientists can counter some of the gender imbalances typical of strongly gender unequal contexts. An open network structure can provide women with privileged access to relevant knowledge and leverage their support from peers for proposed action-plans [10, 28, 29]. Based on the above discussion, we hypothesise that:

**Hypothesis 2:** *Women’s research networks are more likely to display a more open network structure compared to men*.

#### Brokerage roles in professional networks

The joint presence of openness (e.g., spanning structural holes) and diversity (e.g., spanning different professional communities) allows for a detailed analysis of multiple types of brokerage and open triads in personal networks. Triads refer to any subsets of three network actors and the possible ties among them and have been studied extensively in the network research literature [57, 58]. Properties of network structure suggest the idea of open triads where a node *j* (ego) is positioned between two other nodes *i* and z (alters), which are not directly linked to each other.

**Fig 3:**
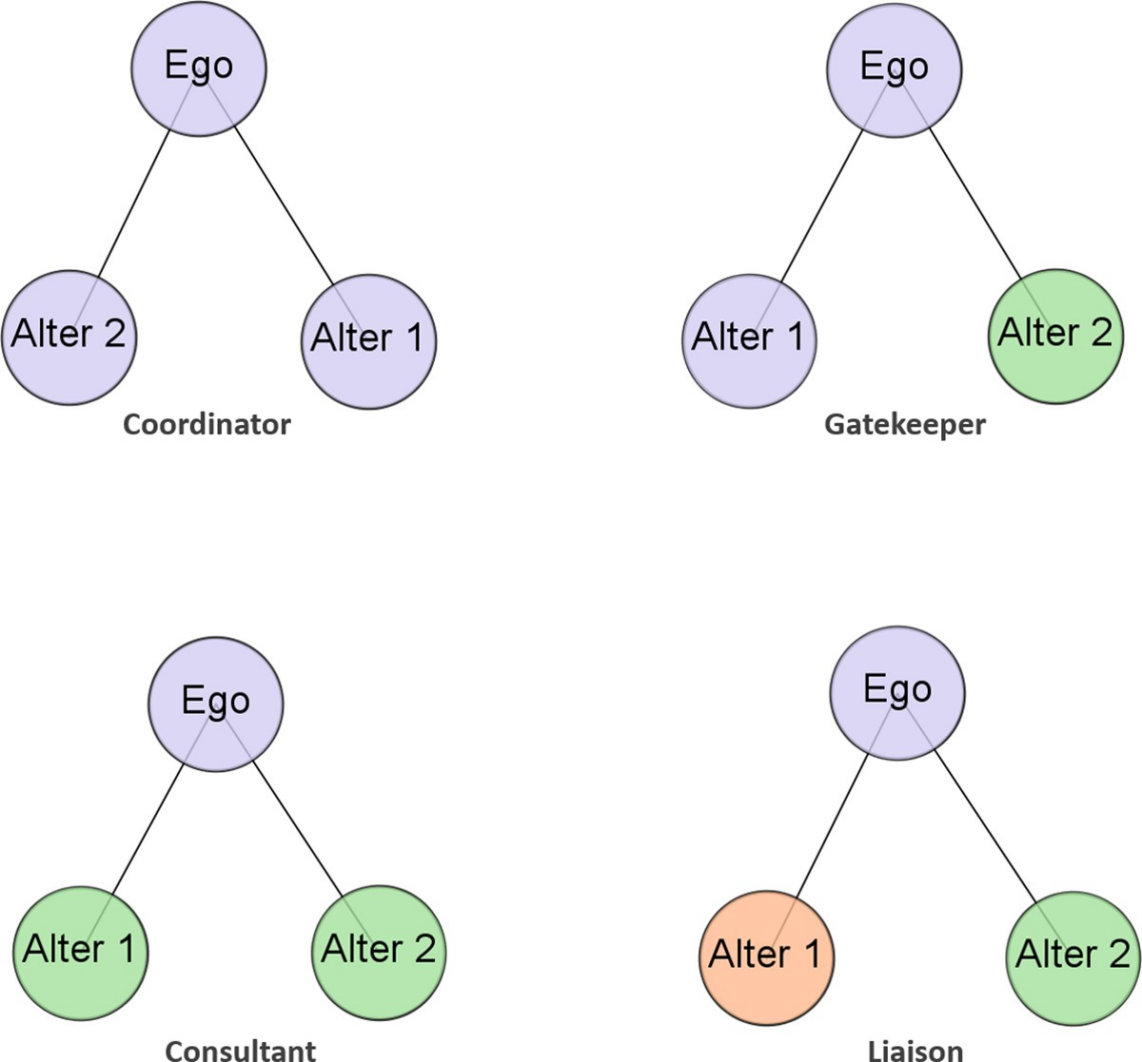
Typology of brokerage roles. Note that Gould and Fernández [3] originally identified five different brokerage roles in the context of directed (bidirectional) ties among the actors. Since we use undirected ties, only four brokerage roles can be distinguished.

Drawing on the network properties of openness and diversity, Gould and Fernández [3] and Fernandez and Gould [4] build on the notion of open triads and Freeman’s [59] idea of betweenness centrality, and propose different types of brokerage roles (Fig 3). For the purposes of our research, we readapted this typology to professional communities. In the “coordinator” type, all three actors belong to the same professional community; “gatekeeper” corresponds to an open triad where the alters belong to different professional communities, one of which is the same as that of the ego; “consultant” includes alters from the same professional community, which is different from that of the ego; and “liaison”, which is the most heterogeneous open triad, is where all three actors belong to different professional communities. Several studies adopt this typology to examine academic inventors [60], brokerage capacity in small and medium sized enterprises [61] and innovation in biotechnology firms [57].

In sum, a personal network configuration that includes brokerage roles is likely to be significant for knowledge generation, since the intermediary role of brokers has the potential to allow access to non-redundant knowledge and diverse research perspectives from heterogeneous professional communities. By looking jointly at diversity and openness in personal networks, we contend that women’s networks are likely to exhibit a wider range of brokerage roles compared to men, since these roles provide new opportunities for knowledge recombination and for greater social legitimacy. Thus, we hypothesise that:

**Hypothesis 3**: *Women’s research networks are more likely to display a broader range of brokerage roles compared to men*.

#### Dissimilarity of brokered partners

Finally, we consider the similarity of brokered partners (alters) vis a vis the focal actor’s (ego) professional community. Not all brokerage roles are the same in terms of cognitive distance from the researcher. They can be ranked from the most "similar" to the ego to the most "distant" from the ego, according to the efforts and costs involved in maintaining and building ties to actors in diverse communities. This ranking was proposed by Jasny and Lubell [62], who suggest that the most similar is the coordinator role because the alters belong to the same group as the ego, and that liaison is the most distant because ego and alters are from entirely different groups. They consider gatekeepers and consultants as between these two extremes, in increasing order of distance from gatekeeper to consultant. This distinction is based on the fact that a consultant role involves alters who belong to a different group than the ego [62].

Although, to our knowledge, there are no studies that analyse the extent to which women form triads involving more dissimilar alters, in terms of institutional or professional community membership, there is some evidence that women’s collaboration strategies involve more heterogeneous networks [6, 22, 39]. For instance, Borrego and Creamer [22] found a significant difference between men’s and women’s collaborations with people from "very different" disciplines from their own. Therefore, based on the rationale for Hypotheses 1, 2 and 3, related to the potential advantages to women associated to the formation of diverse and open networks, we hypothesise that:

**Hypothesis 4**: *Women’s research networks are more likely to include brokerage roles that involve ties to highly dissimilar professional communities compared to men*.

## Data and methods

### Research context and sample

The study context is biomedical research. In the field of biomedicine, research is often based on cooperation among multiple actors–basic scientists, clinical scientists, medical practitioners, patients, among others–and is expected to enhance the flow of knowledge arising from clinical practice questions that guide basic research, and knowledge from theories related to disease pathways that inform clinical practice [63, 64]. Initiatives to encourage cooperation among these multiple biomedical communities are high on the agendas of policy makers trying to increase medical innovations and have been described as “translational” research initiatives (i.e., translation of scientific discoveries to solve real world problems) [65, 66].

We investigate the biomedical research community in Spain. The target population of biomedical scientists is drawn from the Biomedical Research Networking Centres (CIBERs) initiative, which was launched by the Spanish Government in 2007 to promote and increase translational research in biomedicine. This initiative was aimed at enhancing collaboration among research groups in universities, hospitals, research centres and firms, working on similar pathologies. In 2013 when we were conducting our research, there were nine CIBERs: Bioengineering, Biomaterials and Nanomedicine (CIBER-BBN), Diabetes and Metabolic Associated Diseases (CIBER-DEM), Epidemiology and Public Health (CIBER-ESP), Hepatic and Digestive Diseases (CIBER-EHD), Obesity and Nutrition (CIBER-OBN), Mental Health (CIBER-SAM), Neurodegenerative Diseases (CIBER-NED), Rare Diseases (CIBER-ER) and Respiratory Diseases (CIBER-ES). The number of research groups related to each CIBER varies (e.g., CIBER-SAM includes 26 research groups and CIBER-BBN includes 47 research groups). Data on the CIBER populations with regard to the Spanish population of biomedical scientists is provided in S1 Table. Note that our target sample accounts for a substantial proportion of the total population of Spanish biomedical scientists and, since CIBER groups’ eligibility for funding is based on demonstration of research excellence through highly competitive open calls, we expect these scientists to be excellent researchers in their respective fields of biomedical research in Spain.

A CIBER population database was built using information available from the CIBER public directories (https://www.ciberisciii.es/en), which includes personal data and contact information for all CIBER group members and identifies each group’s Principal Investigator (PI). Our research population comprises 4,758 biomedical scientists (i.e. PIs, senior researchers, post-docs and early stage researchers)–our focal actors or egos–plus technicians affiliated to the research groups in the nine CIBERs. This archival data provided the target population for our large-scale survey, from which we have obtained the primary data for this study. In preparation for the survey, we conducted interviews with CIBER scientific directors, research group PIs and other biomedical scientists, during June 2012 to March 2013 in order to obtain information to allow construction of our questionnaire. The resulting survey covers aspects related to the structure and composition of scientists’ personal networks. It also includes a number of attitudinal, motivational and socio-demographic questions. The questionnaire was implemented on Qualtrics and administered in April 2013 to all biomedical scientists in the nine CIBERs. S1 and S2 Files respectively provide the Spanish and English versions of the complete questionnaire. We received 1,309 responses, an overall response rate of 27.5%. Missing values for some of the questions reduced the number of usable responses to 897, an effective response rate of 19%, similar to that achieved by other academic scientist surveys [67]. The distribution of respondents was 30.9% affiliated to a university, 33% to a hospital, 25.9% to a public research institution and 10.2% to a private research body or other similar institution. S2 Table presents the response rates based on respondent’s CIBER affiliation showing that, despite finding some significant differences, the overall distribution of response rates was fairly homogeneous.

### Data analysis

We rely on regression analysis to investigate how gender might influence diversity, openness and the range and type of brokerage roles in personal research networks. We employed an egocentric network approach [1, 68] to capture individual scientists’ critical network contacts. Our respondents were asked to list up to ten individuals outside their research group (i.e., alters), who had been particularly important to their research activities. They were asked also about interactions among alters in the context of their professional activities. The choice of a bounded number of collaborators is standard in surveys related to ego-networks [2] (p. 121–122).

To capture the extent to which an individual’s personal network spans professional communities, respondents were asked to categorise their contacts as basic scientist; clinical scientist; medical practitioner or patient representative; and public administration, industry or other. Table 1 presents the proportion of scientists with at least one tie to each of these four professional communities, comparing between women and men. It can be seen that more than 70% of men’s and women’s personal networks include basic scientists (i.e., at least one tie to this community). The proportion of men with ties to clinical scientists is larger than the proportion of women with ties to that community, but a larger proportion of women than men have at least one tie to a medical practitioner and a patient representative, or to someone working in public administration, industry or some other professional sector.

**Table 1:** Proportion of scientists (by gender) with at least one tie to another professional community.

Alter community	Women	Men
Basic scientists	73.0%	71.5%
Clinical scientists	53.0%	60.0%
Medical practitioners or patient representatives	13.5%	10.2%
Public administration, industry, other	29.0%	25.4%

#### Dependent variables

**Fig 4:**
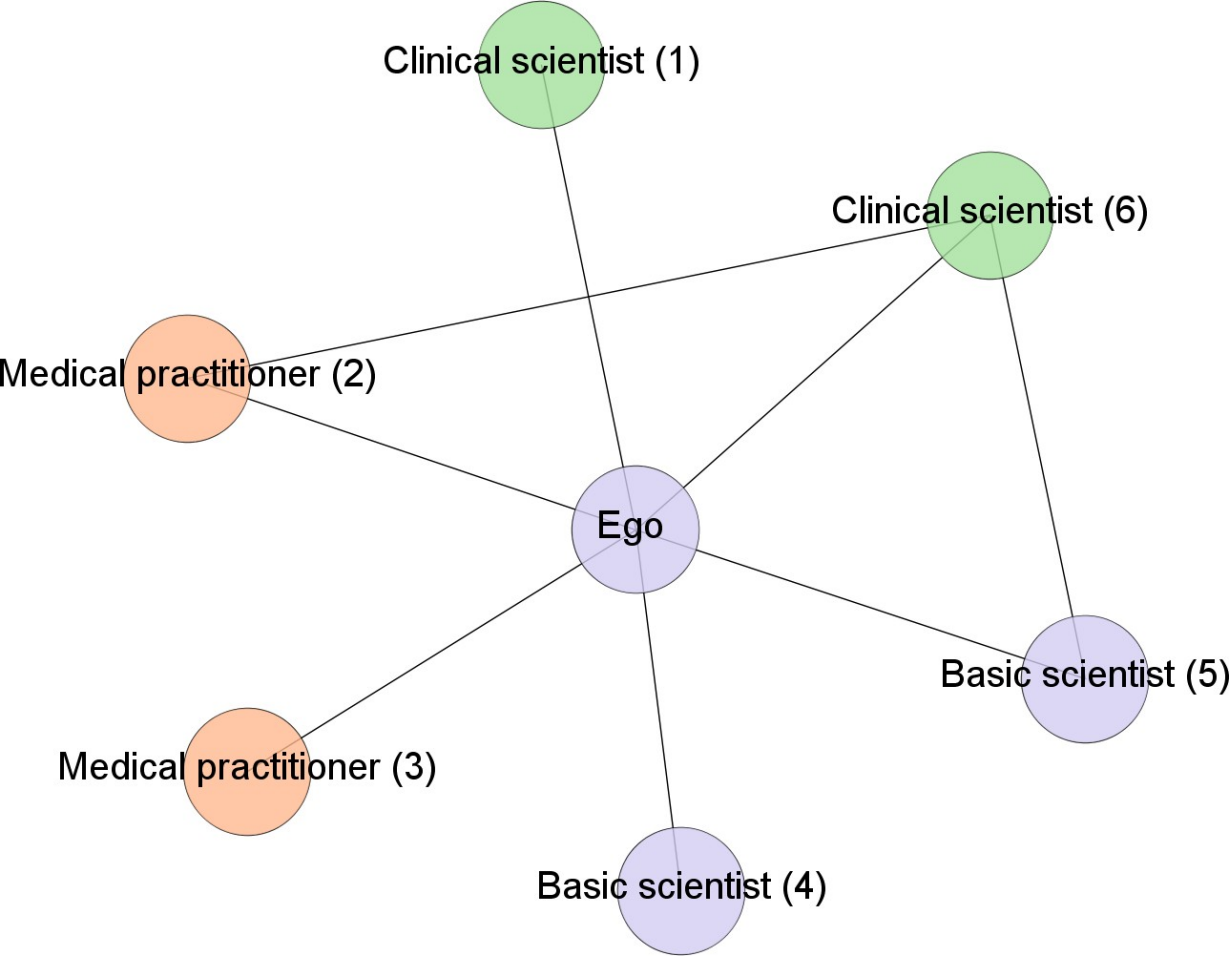
Example of the brokerage roles held by a basic scientist. The total number of brokerage roles held by the scientist is 13, distributed across the categories as follows: No. of coordinator roles = 1 (4–5); No. of gatekeeper roles = 7 (1–4, 1–5, 2–4, 2–5, 3–4, 3–5, 4–6); No. of consultant roles = 2 (1–6, 2–3); No. of liaison roles = 3 (1–2, 1–3, 3–6).

To conduct the empirical analysis, we built five dependent variables (DV) to capture the different network profiles of our sample of biomedical scientists. These variables include:

- DV1 measures network partner diversity (*partner diversity*); we computed a Shannon index [69, 70], which provides a measure of the diversity and evenness in the frequency distribution of the professional communities of network partners (alters). It is calculated as: $H=-\sum_{i=1}^{s}\left(p_{i}){(\ln\;p}_{i}\right)$, where *p*_*i*_ denotes the relative frequency of alters’ professional community groups and *lnp*_*i*_ is the natural logarithm of this ratio;
- DV2 is network openness (*openness*), which is based on the number of structural holes (i.e., absence of alter-alter ties) in the ego network [71], divided by the total number of possible alter-alter ties in the personal network: [*n* (*n*– 1) / 2] [58]. The ratio ranges from 0 to 1, with low values (close to zero) reflecting low openness (i.e., dense/close network) and high values (close to 1) reflecting high openness (i.e., open network). Openness is highest if there are no ties between alters in the ego network;
- DV3 is the *range of brokerage roles*, which we measure using the Shannon index, for the four brokerage roles described in the background section. *Range of brokerage roles* includes information on how many of the four roles are present in the personal network (variety) and the frequency of each brokerage role (evenness). For example, one individual might occupy more than one brokerage role in the research network, but with different frequency for each. Fig 4 depicts the ego-network of a basic scientist in our dataset and shows that a focal actor can occupy multiple brokerage roles;
- to capture brokerage roles involving highly dissimilar partners, we counted the number of times each ego occupied a *consultant* (DV4) or a *liaison* (DV5) role (the most heterogeneous brokerage triad types) in his or her personal research network.

To investigate Hypotheses 1, 2 and 3, we employ Tobit regression, since partner diversity and range of brokerage roles are left-censored variables, whereas openness is bounded at both ends. To ensure conformity to the normality assumption required by a Tobit regression, we log transformed the Shannon index. S3 File provides the QQ-plots to check if the dependent variables display a normal distribution. Note that deviation from this distribution is due mainly to the large number of zeros (lower bound scores) in our variables. To investigate Hypothesis 4, we used a Negative Binomial model since our dependent variables are the respondent’s number of consultant and liaison roles. Given the significant degree of overdispersion, we did not employ a Poisson model.

#### Independent variable and control variables

The main independent variable is gender, which is a dichotomous variable that takes the value 1 for women and 0 for men. Since there are several other factors that might affect the biomedical research network composition, we include control variables at the individual, research group and institutional levels:

- The individual-level control variables include skills breadth (e.g., respondent’s formal training in 9 research areas), network size, basic or applied research, tertius iungens (i.e., behavioural orientation to connecting others) measured on the scale proposed by Obstfeld [72] and respondent age. Being a PI is measured by a dummy variable: since only 10% of respondents are research group PIs, we extended this category to include researchers who had led a research project in the past, but were not the PI of a research group. This provided a more balanced and representative sample and accounts for academic ranking. We control, also, for individual psychological traits, based on Goldberg’s [73] Big Five scale of personality factors (agreeableness, conscientiousness, extraversion, neuroticism and openness), intrinsic and extrinsic motivations [74, 75] and perceived self-efficacy to be creative [76]. Academic performance is measured by the research group PI’s Mean Normalised Citation Score (MNCS) [77] during the period 1998–2010.
- Research group-level controls measure the cohesiveness of the respondent’s CIBER research group and include group network density (actual vs. potential connections) and frequency of ego’s interaction with group members (daily, weekly, monthly, annually). We control also for research group size and share of female researchers in the group. All of these measures are computed based on information on the CIBER research group of affiliation;
- Institutional-level control variables refer to institutional affiliation, which might affect opportunities to act as a broker. We asked the respondents to indicate their institutional affiliation (university, hospital/clinic, public research organisation, other). To account for respondent’s domain, we defined eight dummy variables for scientific field, corresponding to the nine CIBERs.

## Results

### Descriptive statistics

Table 2 presents the descriptive statistics and correlations, for the dependent variables. We observe a medium-high level of openness and a low frequency of consultant or liaison roles (i.e., 17% of scientists in our sample occupied a liaison brokerage role at least once). The correlations among our dependent variables range between a low of 0.14 and a high of 0.66 with most showing values below 0.50. S3 and S4 Tables provide the mean values, definitions and correlations for our full set of variables.

**Table 2:** Descriptive statistics and correlations for the dependent variables.

	Mean	S.D.	Min	Max	Median	DV1	DV2	DV3	DV4
Partner diversity (DV1)	0.38	0.37	0.00	1.33	0.50	1.00			
Openness (DV2)	0.56	0.37	0.00	1.00	0.67	0.36	1.00		
Range of brokerage roles (DV3)	0.30	0.28	0.00	1.20	0.22	0.66	0.55	1.00	
Consultant (DV4)	2.32	6.74	0.00	45.00	0.00	0.14	0.19	0.39	1.00
Liaison (DV5)	1.24	4.27	0.00	45.00	0.00	0.41	0.17	0.43	0.36

N = 897.

**Fig 5:**
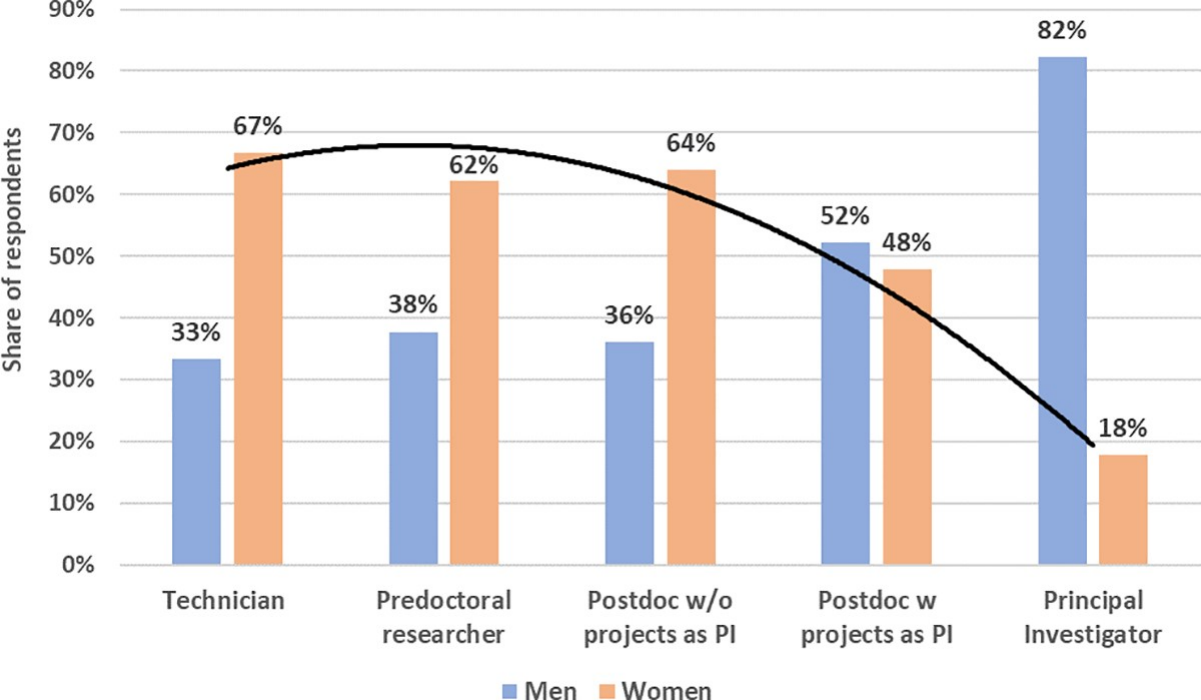
Gender distribution by academic rank. For illustrative purposes, Fig 5 distinguishes between PIs (n = 101) and researchers who had led a research project in the past, but were not the PI of a research group (n = 274). As explained in the methods section, given the low proportion of research groups PIs, we grouped these categories (n = 375) to perform the regression analyses.

Fig 5 depicts the gender distribution among our respondents. The proportion of women (53%, 477) is slightly higher than the share of men (47%, 420). This relative balance disappears if the data are disaggregated by academic rank. In the early stages of a scientific career, women account for over 60% of the cases, but their presence decreases for higher academic positions, with over 82% of the top academic roles (i.e., research group PI, typically professorial level) occupied by men.

**Fig 6:**
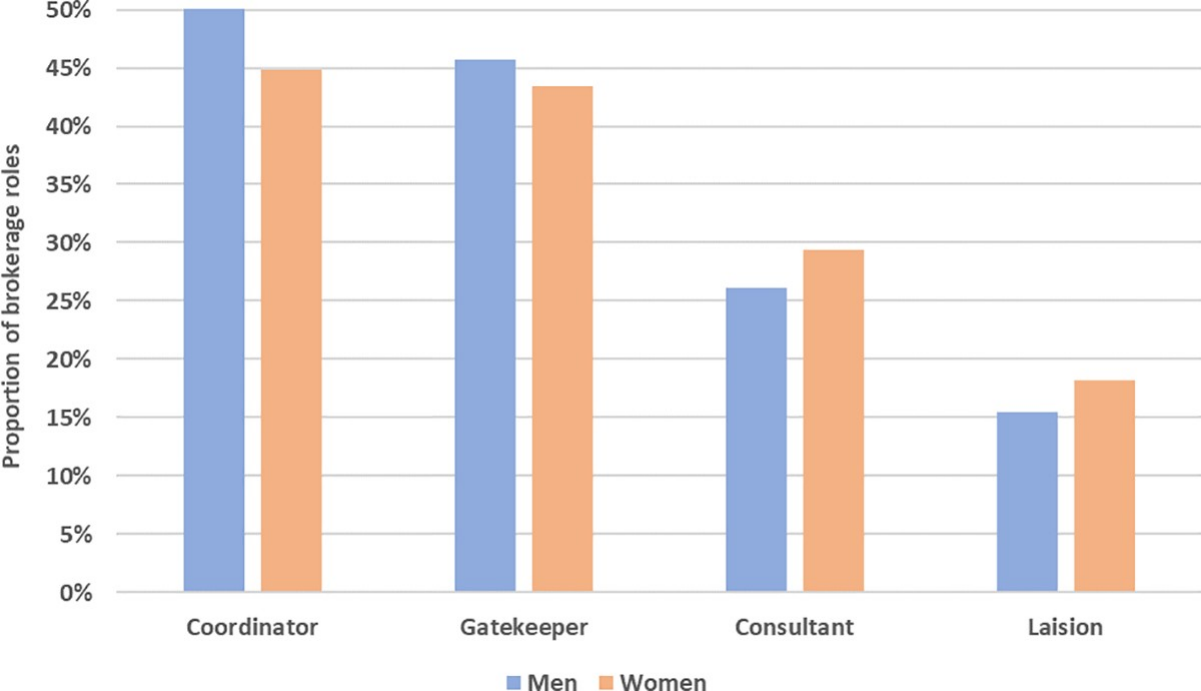
Proportion of brokerage roles held at least once by gender.

Fig 6 is a preliminary view of the relation between gender and brokerage roles in personal research networks. It shows that men tend to form more homogeneous brokerage triads, shown by their higher propensity to occupy a coordinator role, while women more often hold brokerage positions that include cross-boundary ties to dissimilar actors (actors from a different professional community to that of the ego). For instance, 18% of women compared to 15% of men occupied a liaison role at least once (Fig 6). Also, there are no significant differences in the number of contacts (i.e., size of the personal network) between men (4.35) and women (4.18) (χ^2^ = 13.69, p = 0.134), suggesting that the differences are related to network composition, not size.

### Gender influence on diversity of network partners and openness

Table 3 presents the regression results for Hypotheses 1 and 2, which, respectively, test the relationship between gender, and partner diversity and openness. Hypothesis 1 is supported at the 5% significance level (β = 0.086, p-value = 0.040), suggesting that, based on the professional communities of their network alters, women have more diverse networks than men. However, the results for the relationship between being a woman and network openness (β = 0.006, p-value = 0.763) are not statistically significant.

**Table 3:** Results of the Tobit regression models for diversity and openness.

	Model 1			Model 2		
	Partner diversity			Openness		
	*Β*	S.E.	p-value	*β*	S.E.	p-value
*Explanatory variables*						
Woman	**0.086**	**0.042**	**0.040**	0.006	0.020	0.763
*Control variables*						
*Individual level*						
Tertius iungens	**0.074**	**0.021**	**0.000**	-0.006	0.011	0.606
Breadth of skills	-0.002	0.012	0.881	-0.008	0.006	0.171
Principal Investigator	**0.120**	**0.051**	**0.020**	**0.050**	**0.024**	**0.036**
Age	0.000	0.002	0.921	-0.001	0.001	0.574
Conscientiousness	-0.001	0.021	0.950	-0.003	0.010	0.761
Neuroticism	-0.008	0.018	0.649	0.003	0.009	0.766
Openness (personality)	0.005	0.025	0.848	0.020	0.012	0.097
Extraversion	0.012	0.017	0.480	0.013	0.009	0.158
Agreeableness	0.014	0.024	0.569	0.005	0.013	0.684
Intrinsic motivation	-0.032	0.027	0.235	**-0.030**	**0.014**	**0.030**
Extrinsic motivation	0.016	0.018	0.378	-0.005	0.009	0.574
Basic orientation	**-0.115**	**0.039**	**0.004**	0.034	0.022	0.121
Network size	**0.107**	**0.008**	**0.000**	**0.034**	**0.004**	**0.000**
Creative self-efficacy	0.000	0.028	0.995	-0.009	0.015	0.530
MNCS	-0.004	0.017	0.836	0.003	0.007	0.628
*Research Group*						
Group network density	-0.030	0.081	0.710	**-0.080**	**0.040**	**0.047**
Group network frequency	0.001	0.027	0.972	0.020	0.015	0.188
Share of females per group	**-0.003**	**0.001**	**0.042**	0.000	0.001	0.704
Team size	0.000	0.002	0.928	-0.001	0.001	0.374
*Organisational level*						
CIBER dummies		Yes			Yes	
University	0.031	0.049	0.520	0.044	0.026	0.090
Hospital	0.052	0.050	0.299	-0.005	0.027	0.847
Constant	-0.217	0.314	0.489	0.083	0.177	0.640
Cox & Snell pseudo R^2^		0.284			0.127	
N		897			897	

Eight dummy variables accounting for the CIBER domain are included, but not reported in the Table.

### Gender influence on range and type of brokerage roles

Table 4 presents the results of the regression analysis for range and type of brokerages roles. Model 3 tests the hypothesis that women occupy a broader range of brokerage roles. We found that being woman is positively, but not significantly (β = 0.017, p = 0.094) associated to the propensity to hold multiple brokerage positions. This rejects Hypothesis 3.

**Table 4:** Results of the Tobit and negative binomial models.

	Model 3			Model 4			Model 5		
	Range of brokerage roles			Consultant			Liaison		
	*Β*	S.E.	P	*Β*	S.E.	P	*β*	S.E.	P
*Explanatory variables*									
Woman	0.017	0.010	0.094	**0.372**	0.175	**0.033**	**0.479**	0.206	**0.020**
*Control variables*									
*Individual level*									
Tertius iungens	**0.010**	0.005	**0.036**	**0.321**	0.086	**0.000**	0.159	0.124	0.198
Breadth of skills	-0.002	0.003	0.452	**-0.137**	0.041	**0.001**	-0.069	0.056	0.221
Principal Investigator	0.023	0.012	0.051	-0.243	0.166	0.144	0.099	0.236	0.675
Age	0.000	0.001	0.883	0.001	0.008	0.952	0.005	0.013	0.700
Conscientiousness	-0.004	0.005	0.395	0.118	0.076	0.119	0.044	0.095	0.644
Neuroticism	0.002	0.004	0.549	-0.108	0.066	0.103	-0.093	0.099	0.351
Openness (personality)	0.010	0.006	0.098	**0.198**	0.100	**0.047**	0.057	0.119	0.632
Extraversion	0.000	0.005	0.919	-0.020	0.066	0.766	0.031	0.103	0.761
Agreeableness	0.004	0.006	0.500	0.133	0.084	0.116	-0.076	0.125	0.541
Intrinsic motivation	**-0.014**	0.007	**0.030**	**-0.208**	0.098	**0.033**	-0.108	0.139	0.436
Extrinsic motivation	-0.002	0.004	0.662	-0.010	0.070	0.887	-0.109	0.093	0.240
Basic orientation	-0.001	0.010	0.936	0.045	0.140	0.747	**-0.703**	0.202	**0.000**
Network size	**0.076**	0.002	**0.000**	**0.589**	0.034	**0.000**	**0.584**	0.043	**0.000**
Creative self-efficacy	0.001	0.006	0.854	-0.096	0.103	0.348	0.184	0.134	0.169
MNCS	0.002	0.003	0.447	0.030	0.031	0.339	-0.046	0.083	0.580
*Research Group*									
Group network density	**-0.047**	0.019	**0.012**	**-0.563**	0.259	**0.030**	-0.568	0.387	0.142
Group network frequency	0.001	0.007	0.855	-0.165	0.111	0.136	-0.116	0.172	0.502
Share of females per group	0.000	0.000	0.662	0.004	0.005	0.433	-0.008	0.007	0.216
Team size	0.000	0.000	0.320	0.006	0.008	0.426	0.012	0.009	0.156
*Organisational level*									
CIBER dummies		Yes			Yes			Yes	
University	**0.037**	0.012	**0.003**	0.335	0.206	0.103	0.188	0.250	0.453
Hospital	0.016	0.013	0.221	**0.617**	0.200	**0.002**	0.357	0.255	0.161
Constant	-0.100	0.079	0.205	**-4.481**	1.110	**0.000**	-1.467	1.504	0.329
Cox & Snell pseudo R^2^		0.693			0.280			0.157	
N		897			897			897	

Eight dummy variables accounting for the CIBER domain are included, but not reported in the Table.

Models 4 and 5 test Hypothesis 4 in relation to the most heterogeneous brokerage triads. Table 4 shows that, in their corresponding personal research networks, women are more likely than men to occupy consultant and liaison roles. The association between being a woman and a higher number of consultant brokerage roles (i.e., intermediation between two alters from the same community, which is different from that of the ego), is positive and significant at the 5% level (β = 0.372, p-value = 0.033). The effect of being woman and occupying a higher number of liaison brokerage roles (i.e., intermediation between two alters from different professional communities neither of which is common to the ego) is also positive and statistically significant (β = 0.479, p-value 0.020). These results provide strong support for Hypothesis 4. To check the robustness of our models, we used Ordinary Least Squares (OLS): the results were similar (S5 Table).

Finally, we consider whether the relationship between gender and network diversity is contingent on the scientist’s academic status. We found no evidence of a contingent effect of academic status (i.e., interplay between women and PI-role), suggesting that, regardless of academic ranking, women are more likely than men to form networks with more diverse partners and to be involved in several heterogeneous open triads. These results are presented in S6 Table.

## Discussions and conclusions

Work on gender and networks is closely connected to unequal working environments and professional career prospects. In this research, we examined whether network formation patterns display systematic differences by gender.

In the course of their careers, women in science face a range of problems that are different from those faced by men. The metaphor of a leaky pipeline has been used to account for the declining proportion of women in science—from graduate education level to senior academic positions. As suggested by previous research, professional networks can play a critical role in career advancement, since they allow access to information and resources [2], increased scientific productivity [31, 60, 78] and innovation [57, 79]. It has been recognised, also, that network intermediaries can reduce the barriers to collaboration and bridge knowledge gaps [43, 52].

The analysis in this paper on the gender dimension in the formation of biomedical research networks, contributes to these research streams. We found evidence that women form different research networks to those formed by men in biomedical research. Specifically, we found that women’s research networks are more diverse and include more consultant and liaison brokerage roles or triads involving dissimilar network partners. Our results indicate, also, that these patterns hold for all academic ranks. Interestingly, for the propensity for more open networks (Hypothesis 2), and range of brokerage roles (Hypothesis 3), we found no significant differences. The latter result is particularly relevant, since our range of brokerage roles includes coordinator (i.e., alters from the same professional community as the ego) and gatekeeper (i.e., one alter from the ego’s professional community and one from a different professional community), which capture more homogeneous triads. This result, combined with the evidence that women are more likely to form networks that include a higher number of consultant and liaison roles, suggests that women outperform men in relation to formation of highly diverse networks (Hypotheses 1 and 4).

From the perspective of biomedical scientists, our findings suggest that women are more likely to act as knowledge flow intermediaries between different types of actors, e.g., basic and clinical researchers. By establishing networks that include a diversity of partners (from both basic and clinical communities), they are likely to advance both fundamental understanding and practice and, therefore, become active players in translational research initiatives. We would argue that women’s propensity to build more diverse social capital not only increases the opportunities for knowledge recombination but also can reduce the barriers to women’s career progress in the scientific community by enhancing their social legitimacy with peers and academic elites.

### Policy implications

Whether inequalities in academia are jeopardising the potential advantages from the diversity in women’s networks and brokerage positions is an important question, and especially in relation to the field of biomedicine. In most European countries, in their early stage scientific careers, women are better represented in biomedicine compared to other scientific domains, but are affected by the leaky-pipeline phenomenon and are poorly represented in top academic positions.

Our results are particularly relevant in a context where the barriers related to scientific, institutional, cultural and economic factors can prevent scientists’ involvement in translational research activities [80–82]. The literature shows that lack of integrative practices and collaboration among researchers, are major obstacles to translational research. Other barriers include the cultural divide between basic science and medical practice, the fragile infrastructures underpinning collaboration between basic and clinical scientists and the lack of grants to support cooperative working involving clinical and bench scientists [82, 83]. For instance, the testing of new drugs is a long process which consumes vast amounts of resources and the risks involved need to be shared among organisations [83, 84]. Given the need for greater collaboration, the diversity in women’s scientific networks provides several benefits for the advancement of translational research in biomedicine.

The ability to work in a translational research setting requires particular skills and capabilities [80, 81]. Many translational research training programmes emphasise the importance of involvement in multidisciplinary research and an understanding of science as a holistic process—especially in the area of health sciences [81, 85]. Policy makers need to take account of the fact that the coordination and integration efforts involved in collaborations among heterogeneous actors, may not be gender-neutral [85]. Women’s access to scientific information is affected negatively by the smaller number of professional collaborations among women in some scientific areas, and their generally lower status and more junior academic positions [86]. The positive impact on innovation of women in brokerage roles is likely to be reduced by the small presence of women in decision-making positions in institutional scientific settings. Although academic status seems to matter less in the context of heterogeneous networks, involving different professional spheres, it can condition the professional impact of the social collaborative structures formed by women in science.

### Directions for future research

We identify the following research avenues. Although our results support the findings in previous work on patterns of collaboration from a gender perspective, that in a scientific research context the “strategic” networking behaviours of women and men differ [18, 22, 34], we suggest caution when interpreting the results of the present research. Granovetter’s [48] theory of weak ties states that although people in socially disadvantaged positions might find strong ties more beneficial, they are forced to fall back on weak ties. Our research reveals some broad patterns based on aggregate data, but in the absence of complementary qualitative interviews with scientists, we cannot make conclusions about the underlying reasons for these patterns. While some studies identify the role of strategic interests in networking, some networking patterns may be a result of certain preferences that can be difficult to isolate from gendered socialisation and role performance. Preferences related to ties to certain actors, such as civil associations, might explain the gender differences related to homophilic networks. For instance, men have been shown to be more likely to establish connections to business [87, 88], whereas women often see civil society organisations as more important for their innovation related activities [89]. Likewise, women working on innovation are more likely to interact with lower status and less powerful actors [7, 28]. This pattern of engagement with a wider variety of, perhaps, more peripheral actors could be interpreted as women’s preference for an intermediary role. In turn, this supposed preference might be largely influenced by other actor’s explicit or implicit demands. For instance, some researchers argue for a legitimacy deficit as central to understanding differences in networking behaviour [29, 36]. Since women are underrepresented in positions of organizational authority (e.g., 18% of research group leaders in our sample were women), they suffer from disadvantages linked to minorities such as being under greater performance pressure than their direct peers or failure to challenge distorted expectations and stereotypes [90]. Schoen et al. [36] show that when the proportion of women in authority positions is high, they benefit from the same network structures as men. This might suggest that women act differently in their attempt to achieve greater social legitimacy (e.g., a successful research career), rather than that they have different preferences.

This research has some limitations. First, our data refer to the biomedical context in Spain; future work could explore whether our findings are generalisable to other research settings and countries. Although an overall shift towards dominance of teamwork in the production of knowledge has been observed in science, there remain important differences related to network size and collaboration practices across different scientific domains [91]. Biomedical research relies on lab scale and capital-intensive equipment, but not to the same extent as physics or other big science related domains; while in mathematics, networks typically take the form of small researcher groups [30, 78, 92]. Moreover, bringing together scientists from different specialities and settings (as in the case of translation research) is frequent in biomedical research, but is not a common feature of all research domains. Second, our cross-sectional data make it difficult to identify direct causal relationships. Our analysis is confined to identifying statistical associations among the key variables. Our findings show that, regardless of their academic position, women forge a greater variety of links to actors from different professional communities. Future work could investigate the contingencies related to this phenomenon. The patterns of network formation might be driven by both unequal environments and personal preferences and gendered socialisation. The individual and combined effects of these aspects are unclear; distinguishing between strategic and social factors might provide more information on the effects of gender on innovation performance. Certain institutional aspects of the innovation system are more feminised/masculinised, for example, administration/industry. A gender-to-gender approach that also takes account of professional diversity, might shed new light on the observed tendencies related to brokerage. Finally, future work linking brokerage position to innovation, could consider the gender aspects highlighted by the present study.

## Supporting information

S1 Table Personnel devoted in 2013 to R&D in medical sciences in Spain. (DOCX)

S2 Table Response rate by CIBER. (DOCX)

S3 Table Variables definitions and mean values by gender. (DOCX)

S4 Table Correlations for the complete set of variables (n = 897). (DOCX)

S5 Table Results of the Tobit regression models for diversity, openness and the range of brokerage roles. (DOCX)

S6 Table Results for the regressions including the interplay between gender and PI. (DOCX)

S1 File Survey to biomedical research networking centres (Spanish). (PDF)

S2 File Survey to biomedical research networking centres (English). (PDF)

S3 File QQ-plots for partner diversity (DV1), openness (DV2) and range of brokerage roles (DV3). (DOCX)
