# Correction: Do women in science form more diverse research networks than men? An analysis of Spanish biomedical scientists

**DOI:** 10.1371/journal.pone.0244622

**Published:** 2020-12-21

**Authors:** Adrián A. Díaz-Faes, Paula Otero-Hermida, Müge Ozman, Pablo D’Este

There is an error in affiliation 2 for author Müge Ozman. The correct affiliation 2 is: Université Paris-Saclay, LITEM, 91025, Evry-Courcouronnes, France
