# Supplementary material for: Do women in science form more diverse research networks than men? An analysis of Spanish biomedical scientists
Source: PLoS One. 2020 Aug 27;15(8):e0238229. doi: 10.1371/journal.pone.0238229 (PMC7451541; doi:10.1371/journal.pone.0238229)
Supplement: S1 Table — (DOCX) [file pone.0238229.s001.docx]

|  | Universities | Public Administration | NGOs | Total |
| --- | --- | --- | --- | --- |
| Total personnel in R&D (number of persons) | 24,974 | 23,776 | 406 | 49,156 |
| Personnel in R&D: researchers | **20,120** | **16,093** | **290** | **36,503** |
| Women in R&D (number of persons) | 11,489 | 13,974 | 237 | 25,700 |
| Women in R&D: researchers | **8,728** | **8,701** | **158** | **17,587** |
| Doctors | 12,771 | 6,244 | 221 | 19,236 |

**S1 Table. Personnel devoted in 2013 to R&D in medical sciences in Spain.**

Note: data from the *2013 R&D Statistics* published by the Spanish National Statistics Institute (https://www.ine.es/) (INE, 2013).
