# Supplementary material for: Do women in science form more diverse research networks than men? An analysis of Spanish biomedical scientists
Source: PLoS One. 2020 Aug 27;15(8):e0238229. doi: 10.1371/journal.pone.0238229 (PMC7451541; doi:10.1371/journal.pone.0238229)
Supplement: S2 Table — (DOCX) [file pone.0238229.s002.docx]

**S2 Table. Response rate by CIBER.**

| CIBER | Submitted | Share by CIBER (%) | Valid responses | Valid-sample response rate (%) | Complete responses | Complete-sample response rate (%) |
| --- | --- | --- | --- | --- | --- | --- |
|  | (1) | (2) | (3) | (3) / (1) | (4) | (4) / (1) |
| BBN | 872 | 18.3 | 238 | 27.3 | 162 | 18.5 |
| DEM | 331 | 7.0 | 96 | 29.0 | 68 | 20.5 |
| EHD | 459 | 9.6 | 154 | 33.6^*^ | 110 | 24.0 |
| ER | 517 | 10.9 | 177 | 34.2^*^ | 129 | 24.9 |
| ES | 439 | 9.2 | 159 | 36.2^*^ | 114 | 26.0 |
| ESP | 610 | 12.8 | 107 | 17.5^*^ | 67 | 11.0 |
| NED | 750 | 15.8 | 186 | 24.8 | 126 | 16.8 |
| OBN | 303 | 6.4 | 71 | 23.4 | 46 | 15.2 |
| SAM | 477 | 10.0 | 121 | 25.4 | 75 | 15.7 |
| Total | 4,758 | 100% | 1,309 | 27.5 | 897 | 18.9 |

Note: ^*^ indicates significant statistical difference in response rates (p < 0.05).
