# Supplementary material for: Do women in science form more diverse research networks than men? An analysis of Spanish biomedical scientists
Source: PLoS One. 2020 Aug 27;15(8):e0238229. doi: 10.1371/journal.pone.0238229 (PMC7451541; doi:10.1371/journal.pone.0238229)
Supplement: S3 Table — (DOCX) [file pone.0238229.s003.docx]

**S3 Table. Variables definitions and mean values by gender.**

|  | **Variables** | **Definition** | **Total sample** | | | | **Woman** | **Man** |
| --- | --- | --- | --- | --- | --- | --- | --- | --- |
|  |  |  | **Mean** | **S.D** | **Min** | **Max** | **Mean** | **Mean** |
| **Dependent variables** | Partner diversity | Shannon index of the diversity of network partners | 0.38 | 0.37 | 0.00 | 1.33 | 0.38 | 0.37 |
|  | Openness | Actual vs potential connections of the ego’s research network | 0.56 | 0.37 | 0.00 | 1.00 | 0.56 | 0.56 |
|  | Range of brokerage roles | Shannon index of the range of brokerage roles held by focal actors | 0.30 | 0.28 | 0.00 | 1.20 | 0.32 | 0.29 |
|  | Consultant | Number of consultant position held by focal actors | 2.32 | 6.74 | 0.00 | 45.00 | 2.49 | 2.14 |
|  | Liaison | Number of liaison positions held by focal actors | 1.24 | 4.27 | 0.00 | 45.00 | 1.29 | 1.19 |
| **Individual level** | Tertius iungens | Orientation towards connecting people | 5.16 | 1.17 | 1.00 | 7.00 | 5.07 | 5.27 |
|  | Breadth of skills | Respondents’ formal training in nine areas | 2.92 | 1.85 | 0.00 | 9.00 | 2.84 | 3.01 |
|  | Principal Investigator | Whether the focal actor is a women or not | 0.42 | 0.49 | 0.00 | 1.00 | 0.31 | 0.54 |
|  | Age | Age of the respondent | 42.46 | 10.53 | 23.00 | 78.00 | 39.80 | 45.49 |
|  | Conscientiousness | Big Five personality traits | 5.62 | 0.98 | 2.25 | 7.00 | 5.77 | 5.45 |
|  | Neuroticism |  | 3.36 | 1.06 | 1.00 | 7.00 | 3.41 | 3.30 |
|  | Openness (personality) |  | 5.39 | 0.98 | 1.00 | 7.00 | 5.31 | 5.48 |
|  | Extraversion |  | 4.00 | 1.14 | 1.00 | 7.00 | 4.11 | 3.88 |
|  | Agreeableness |  | 5.73 | 0.87 | 2.00 | 7.00 | 5.89 | 5.55 |
|  | Intrinsic motivation | Motivations scales | 6.19 | 0.79 | 1.00 | 7.00 | 6.24 | 6.13 |
|  | Extrinsic motivation |  | 3.70 | 1.18 | 1.00 | 7.00 | 3.56 | 3.87 |
|  | Basic orientation | Whether the focal actor is involved in basic or applied research | 2.49 | 0.52 | 1.00 | 3.00 | 2.46 | 2.53 |
|  | Network size | Relevant contact outside the research group | 4.26 | 2.56 | 1.00 | 10.00 | 4.18 | 4.35 |
|  | Creative self-efficacy | Perceived creative self-efficacy scale | 5.24 | 0.92 | 1.33 | 7.00 | 5.14 | 5.35 |
|  | MNCS | Mean normalized citation score | 0.99 | 1.47 | 0.00 | 28.63 | 0.86 | 1.13 |
| **Research group level** | Group network density | Frequency of ego’s interaction with group members | 0.71 | 0.26 | 0.00 | 1.00 | 0.74 | 0.68 |
|  | Group network frequency | Percentage of women within the research group | 2.65 | 0.66 | 1.00 | 4.00 | 2.67 | 2.63 |
|  | Share of females per group | Number of female group members | 56.56 | 16.17 | 18.18 | 100.00 | 60.81 | 51.73 |
|  | Team size | Number of group member | 18.08 | 10.56 | 4.00 | 79.00 | 17.70 | 18.50 |
| **Institutional level** | BBN | CIBER domain | 0.18 | 0.38 | 0.00 | 1.00 | 0.18 | 0.18 |
|  | DEM |  | 0.08 | 0.26 | 0.00 | 1.00 | 0.07 | 0.08 |
|  | EHD |  | 0.12 | 0.33 | 0.00 | 1.00 | 0.10 | 0.15 |
|  | ER |  | 0.14 | 0.35 | 0.00 | 1.00 | 0.17 | 0.11 |
|  | ES |  | 0.13 | 0.33 | 0.00 | 1.00 | 0.12 | 0.14 |
|  | ESP |  | 0.07 | 0.26 | 0.00 | 1.00 | 0.07 | 0.08 |
|  | NED |  | 0.14 | 0.35 | 0.00 | 1.00 | 0.14 | 0.14 |
|  | OBN |  | 0.05 | 0.22 | 0.00 | 1.00 | 0.04 | 0.06 |
|  | University | Ego's affiliation | 0.31 | 0.46 | 0.00 | 1.00 | 0.31 | 0.31 |
|  | Hospital |  | 0.33 | 0.47 | 0.00 | 1.00 | 0.29 | 0.38 |

Note: N = 897 (Women = 477, Men = 420). Given the low proportion of PIs, Principal Investigator category was extended to include researchers who had led a research project in the past, but were not the PI of a research group.
