# Supplementary material for: Do women in science form more diverse research networks than men? An analysis of Spanish biomedical scientists
Source: PLoS One. 2020 Aug 27;15(8):e0238229. doi: 10.1371/journal.pone.0238229 (PMC7451541; doi:10.1371/journal.pone.0238229)
Supplement: S4 Table — (DOCX) [file pone.0238229.s004.docx]

**S4 Table. Correlations for the complete set of variables (n = 897).**

|  |  | 1 | 2 | 3 | 4 | 5 | 6 | 7 | 8 | 9 | 10 | 11 | 12 | 13 | 14 | 15 | 16 | 17 | 18 |
| --- | --- | --- | --- | --- | --- | --- | --- | --- | --- | --- | --- | --- | --- | --- | --- | --- | --- | --- | --- |
| 1 | Partner diversity | 1.00 |  |  |  |  |  |  |  |  |  |  |  |  |  |  |  |  |  |
| 2 | Openness | 0.36 | 1.00 |  |  |  |  |  |  |  |  |  |  |  |  |  |  |  |  |
| 3 | Range of brokerage roles | 0.66 | 0.55 | 1.00 |  |  |  |  |  |  |  |  |  |  |  |  |  |  |  |
| 4 | Consultant | 0.14 | 0.19 | 0.39 | 1.00 |  |  |  |  |  |  |  |  |  |  |  |  |  |  |
| 5 | Liaison | 0.41 | 0.17 | 0.43 | 0.36 | 1.00 |  |  |  |  |  |  |  |  |  |  |  |  |  |
| 6 | Tertius iungens | 0.25 | 0.09 | 0.27 | 0.13 | 0.11 | 1.00 |  |  |  |  |  |  |  |  |  |  |  |  |
| 7 | Breadth of skills | 0.12 | 0.03 | 0.17 | 0.02 | 0.05 | 0.23 | 1.00 |  |  |  |  |  |  |  |  |  |  |  |
| 8 | Principal Investigator | 0.18 | 0.15 | 0.21 | 0.03 | 0.06 | 0.18 | 0.10 | 1.00 |  |  |  |  |  |  |  |  |  |  |
| 9 | Age | 0.13 | 0.10 | 0.17 | 0.06 | 0.04 | 0.13 | 0.07 | 0.57 | 1.00 |  |  |  |  |  |  |  |  |  |
| 10 | Conscientiousness | -0.03 | -0.07 | -0.08 | -0.02 | -0.04 | 0.08 | 0.05 | -0.15 | -0.10 | 1.00 |  |  |  |  |  |  |  |  |
| 11 | Neuroticism | -0.03 | -0.01 | -0.04 | -0.07 | 0.00 | -0.04 | 0.02 | 0.02 | 0.03 | -0.10 | 1.00 |  |  |  |  |  |  |  |
| 12 | Openness (personality) | 0.08 | 0.08 | 0.14 | 0.08 | 0.02 | 0.26 | 0.06 | 0.02 | -0.04 | 0.00 | -0.14 | 1.00 |  |  |  |  |  |  |
| 13 | Extraversion | 0.10 | 0.07 | 0.10 | 0.02 | 0.04 | 0.26 | 0.16 | -0.05 | -0.14 | -0.01 | -0.08 | 0.18 | 1.00 |  |  |  |  |  |
| 14 | Agreeableness | 0.06 | 0.04 | 0.05 | 0.03 | -0.02 | 0.16 | 0.09 | -0.08 | -0.09 | 0.16 | -0.06 | 0.28 | 0.21 | 1.00 |  |  |  |  |
| 15 | Intrinsic motivation | 0.02 | -0.03 | 0.03 | 0.02 | -0.02 | 0.29 | 0.04 | 0.05 | -0.08 | 0.13 | -0.05 | 0.24 | 0.08 | 0.15 | 1.00 |  |  |  |
| 16 | Extrinsic motivation | 0.03 | -0.04 | 0.00 | -0.01 | -0.03 | 0.23 | 0.05 | 0.11 | 0.11 | 0.05 | 0.01 | -0.04 | 0.10 | -0.04 | 0.24 | 1.00 |  |  |
| 17 | Basic orientation | -0.06 | 0.05 | 0.02 | 0.01 | -0.08 | 0.24 | 0.03 | 0.07 | 0.00 | 0.06 | -0.05 | 0.15 | 0.00 | -0.01 | 0.20 | 0.12 | 1.00 |  |
| 18 | Network size | 0.48 | 0.40 | 0.85 | 0.46 | 0.36 | 0.26 | 0.18 | 0.20 | 0.16 | -0.07 | -0.05 | 0.11 | 0.11 | 0.05 | 0.04 | -0.01 | 0.03 | 1.00 |
| 19 | Creative self-efficacy | 0.12 | 0.03 | 0.16 | 0.08 | 0.10 | 0.50 | 0.18 | 0.08 | 0.03 | 0.11 | -0.11 | 0.43 | 0.23 | 0.16 | 0.31 | 0.22 | 0.21 | 0.15 |
| 20 | MNCS | 0.00 | 0.04 | 0.00 | -0.03 | -0.03 | 0.09 | 0.03 | 0.18 | 0.10 | -0.04 | -0.06 | -0.03 | 0.01 | 0.03 | 0.01 | 0.09 | 0.05 | 0.00 |
| 21 | Group network density | -0.06 | -0.09 | -0.11 | -0.04 | -0.03 | -0.06 | 0.00 | -0.10 | -0.08 | 0.10 | 0.02 | -0.01 | -0.02 | -0.01 | 0.03 | -0.01 | 0.00 | -0.06 |
| 22 | Group network frequency | -0.04 | 0.01 | -0.09 | -0.05 | -0.06 | -0.10 | -0.07 | -0.01 | 0.02 | -0.04 | -0.06 | -0.04 | -0.09 | -0.03 | -0.09 | -0.03 | -0.11 | -0.09 |
| 23 | Share of females per group | -0.03 | 0.03 | 0.01 | 0.02 | -0.05 | -0.03 | 0.02 | -0.01 | 0.02 | 0.02 | 0.01 | -0.03 | 0.01 | 0.07 | 0.09 | 0.02 | 0.02 | 0.02 |
| 24 | Team size | -0.03 | -0.01 | -0.05 | 0.03 | 0.01 | -0.08 | -0.17 | -0.08 | -0.19 | -0.13 | -0.03 | -0.03 | 0.00 | -0.04 | -0.08 | -0.03 | -0.01 | -0.04 |
| 25 | BBN | -0.02 | 0.00 | -0.04 | 0.06 | 0.04 | -0.02 | -0.30 | -0.12 | -0.16 | -0.03 | 0.00 | 0.03 | -0.05 | -0.04 | -0.03 | -0.01 | -0.01 | -0.03 |
| 26 | DEM | -0.05 | 0.02 | 0.02 | -0.04 | -0.04 | 0.00 | 0.09 | 0.00 | 0.03 | 0.03 | 0.01 | 0.00 | -0.04 | -0.06 | 0.04 | 0.00 | 0.04 | 0.00 |
| 27 | EHD | 0.02 | 0.03 | 0.04 | 0.02 | 0.00 | 0.07 | 0.12 | 0.14 | 0.13 | -0.01 | -0.02 | -0.04 | 0.02 | -0.04 | -0.01 | 0.09 | -0.01 | 0.05 |
| 28 | ER | 0.11 | 0.06 | 0.07 | -0.01 | 0.02 | 0.07 | 0.02 | 0.03 | 0.06 | 0.02 | 0.05 | 0.01 | -0.03 | 0.02 | 0.09 | -0.06 | -0.03 | 0.06 |
| 29 | ES | -0.02 | -0.07 | -0.05 | -0.04 | -0.01 | -0.01 | 0.05 | 0.02 | 0.08 | 0.03 | 0.02 | -0.08 | 0.02 | -0.03 | -0.01 | 0.00 | 0.01 | -0.05 |
| 30 | ESP | 0.01 | -0.02 | 0.01 | 0.08 | 0.03 | -0.05 | -0.03 | -0.04 | 0.02 | -0.07 | -0.02 | 0.02 | -0.01 | 0.00 | -0.09 | 0.07 | -0.09 | 0.02 |
| 31 | NED | -0.10 | -0.02 | -0.07 | -0.06 | -0.08 | -0.06 | 0.04 | -0.06 | -0.12 | 0.02 | 0.02 | 0.03 | 0.04 | 0.08 | 0.01 | -0.09 | 0.11 | -0.05 |
| 32 | OBN | 0.01 | -0.05 | -0.02 | -0.05 | -0.02 | 0.03 | 0.03 | 0.03 | 0.07 | 0.03 | -0.06 | 0.05 | 0.02 | 0.06 | 0.01 | 0.03 | -0.01 | -0.03 |
| 33 | University | -0.04 | 0.05 | -0.01 | -0.01 | -0.01 | -0.02 | -0.15 | 0.01 | -0.03 | -0.06 | 0.07 | 0.01 | -0.03 | -0.06 | 0.07 | 0.08 | 0.06 | -0.05 |
| 34 | Hospital | 0.11 | -0.01 | 0.08 | 0.06 | 0.04 | 0.05 | 0.19 | 0.12 | 0.19 | 0.03 | 0.01 | -0.07 | 0.02 | -0.02 | -0.08 | 0.05 | -0.19 | 0.08 |

|  |  | 19 | 20 | 21 | 22 | 23 | 24 | 25 | 26 | 27 | 28 | 29 | 30 | 31 | 32 | 33 | 34 |
| --- | --- | --- | --- | --- | --- | --- | --- | --- | --- | --- | --- | --- | --- | --- | --- | --- | --- |
| 19 | Creative self-efficacy | 1.00 |  |  |  |  |  |  |  |  |  |  |  |  |  |  |  |
| 20 | MNCS | 0.06 | 1.00 |  |  |  |  |  |  |  |  |  |  |  |  |  |  |
| 21 | Group network density | -0.04 | -0.04 | 1.00 |  |  |  |  |  |  |  |  |  |  |  |  |  |
| 22 | Group network frequency | -0.10 | 0.01 | -0.06 | 1.00 |  |  |  |  |  |  |  |  |  |  |  |  |
| 23 | Share of females per group | -0.03 | -0.03 | 0.03 | -0.05 | 1.00 |  |  |  |  |  |  |  |  |  |  |  |
| 24 | Team size | -0.03 | -0.05 | -0.20 | 0.12 | -0.08 | 1.00 |  |  |  |  |  |  |  |  |  |  |
| 25 | BBN | 0.01 | -0.05 | -0.09 | 0.08 | -0.13 | 0.37 | 1.00 |  |  |  |  |  |  |  |  |  |
| 26 | DEM | 0.02 | 0.00 | -0.03 | 0.00 | 0.07 | 0.00 | -0.13 | 1.00 |  |  |  |  |  |  |  |  |
| 27 | EHD | 0.02 | 0.20 | 0.03 | 0.03 | -0.16 | -0.19 | -0.18 | -0.11 | 1.00 |  |  |  |  |  |  |  |
| 28 | ER | 0.00 | -0.06 | -0.02 | -0.05 | 0.23 | -0.15 | -0.19 | -0.12 | -0.15 | 1.00 |  |  |  |  |  |  |
| 29 | ES | -0.02 | -0.01 | 0.06 | 0.03 | -0.09 | -0.11 | -0.18 | -0.11 | -0.14 | -0.16 | 1.00 |  |  |  |  |  |
| 30 | ESP | -0.04 | -0.02 | 0.04 | 0.01 | 0.00 | -0.08 | -0.13 | -0.08 | -0.11 | -0.12 | -0.11 | 1.00 |  |  |  |  |
| 31 | NED | 0.00 | 0.02 | 0.03 | -0.12 | 0.00 | 0.01 | -0.19 | -0.12 | -0.15 | -0.17 | -0.15 | -0.11 | 1.00 |  |  |  |
| 32 | OBN | 0.08 | -0.04 | -0.04 | -0.02 | 0.04 | 0.02 | -0.11 | -0.07 | -0.09 | -0.10 | -0.09 | -0.07 | -0.09 | 1.00 |  |  |
| 33 | University | 0.02 | -0.07 | -0.02 | -0.01 | -0.03 | 0.14 | 0.29 | 0.04 | -0.11 | -0.12 | -0.15 | -0.09 | 0.02 | 0.10 | 1.00 |  |
| 34 | Hospital | -0.03 | 0.04 | 0.02 | 0.09 | -0.09 | -0.09 | -0.27 | -0.07 | 0.17 | 0.03 | 0.24 | -0.06 | -0.14 | 0.01 | -0.47 | 1.00 |
