# Supplementary material for: Do women in science form more diverse research networks than men? An analysis of Spanish biomedical scientists
Source: PLoS One. 2020 Aug 27;15(8):e0238229. doi: 10.1371/journal.pone.0238229 (PMC7451541; doi:10.1371/journal.pone.0238229)
Supplement: S5 Table — (DOCX) [file pone.0238229.s005.docx]

**S5 Table. Results of the OLS regression models for partner diversity, openness and the range of brokerage roles.**

|  | **Model 1**  **Partner diversity** | | | **Model 2**  **Openness** | | | **Model 3**  **Range of brokerage roles** | | |
| --- | --- | --- | --- | --- | --- | --- | --- | --- | --- |
|  | ***β*** | **S.E.** | **p** | ***β*** | **S.E.** | **p** | ***β*** | **S.E.** | **p** |
| *Explanatory variables* |  |  |  |  |  |  |  |  |  |
| Woman | 0.046 | 0.023 | **0.049** | 0.017 | 0.025 | 0.500 | 0.014 | 0.008 | 0.081 |
| *Control variables* |  |  |  |  |  |  |  |  |  |
| *Individual level* |  |  |  |  |  |  |  |  |  |
| Tertius iungens | 0.041 | 0.011 | **0.000** | -0.004 | 0.013 | 0.776 | 0.008 | 0.004 | **0.030** |
| Breadth of skills | 0.000 | 0.007 | 0.944 | -0.010 | 0.007 | 0.158 | -0.001 | 0.002 | 0.687 |
| Principal Investigator | 0.065 | 0.030 | **0.027** | 0.057 | 0.029 | **0.046** | 0.015 | 0.009 | 0.105 |
| Age | 0.000 | 0.001 | 0.881 | 0.000 | 0.001 | 0.927 | 0.000 | 0.000 | 0.896 |
| Conscientiousness | -0.002 | 0.012 | 0.884 | -0.005 | 0.012 | 0.665 | -0.003 | 0.004 | 0.439 |
| Neuroticism | -0.006 | 0.010 | 0.545 | 0.006 | 0.011 | 0.586 | 0.001 | 0.003 | 0.726 |
| Openness (personality) | 0.003 | 0.013 | 0.846 | 0.021 | 0.014 | 0.144 | 0.008 | 0.005 | 0.072 |
| Extraversion | 0.004 | 0.009 | 0.655 | 0.014 | 0.011 | 0.206 | -0.002 | 0.004 | 0.617 |
| Agreeableness | 0.007 | 0.013 | 0.624 | 0.009 | 0.015 | 0.525 | 0.002 | 0.005 | 0.716 |
| Intrinsic motivation | -0.018 | 0.015 | 0.243 | -0.034 | 0.017 | **0.042** | -0.010 | 0.005 | 0.063 |
| Extrinsic motivation | 0.008 | 0.010 | 0.457 | -0.006 | 0.011 | 0.550 | -0.002 | 0.003 | 0.588 |
| Basic orientation | -0.065 | 0.022 | **0.004** | 0.042 | 0.026 | 0.103 | -0.004 | 0.008 | 0.640 |
| Network size | 0.062 | 0.005 | **0.000** | 0.057 | 0.005 | **0.000** | 0.066 | 0.002 | **0.000** |
| Creative self-efficacy | 0.005 | 0.015 | 0.727 | -0.013 | 0.017 | 0.449 | 0.004 | 0.005 | 0.459 |
| *Research Group* |  |  |  |  |  |  |  |  |  |
| Group network density | -0.024 | 0.045 | 0.588 | -0.083 | 0.049 | 0.089 | -0.043 | 0.015 | **0.004** |
| Group network frequency | -0.005 | 0.015 | 0.743 | 0.028 | 0.018 | 0.116 | -0.003 | 0.006 | 0.595 |
| Share of females per group | -0.002 | 0.001 | **0.049** | 0.000 | 0.001 | 0.569 | 0.000 | 0.000 | 0.576 |
| Team size | 0.000 | 0.001 | 0.994 | -0.001 | 0.001 | 0.490 | 0.000 | 0.000 | 0.332 |
| MNCS | -0.004 | 0.010 | 0.713 | 0.009 | 0.007 | 0.216 | 0.000 | 0.003 | 0.890 |
| *Organisational level* |  | | |  | | |  | | |
| CIBER dummies | Yes | | | Yes | | | Yes | | |
| University | 0.011 | 0.028 | 0.700 | 0.053 | 0.031 | 0.085 | 0.028 | 0.010 | **0.007** |
| Hospital | 0.026 | 0.030 | 0.377 | -0.008 | 0.031 | 0.802 | 0.016 | 0.011 | 0.155 |
| Constant | 0.173 | 0.177 | 0.330 | -0.156 | 0.209 | 0.456 | -0.021 | 0.062 | 0.732 |
| Cox & Snell pseudo R^2^ | 0.285 | | | 0.201 | | | 0.735 | | |
| N | 897 | | | 897 | | | 897 | | |

Note: To investigate Model 1, Model 2 and Model 3 a Tobit regression is used
