# Supplementary material for: Do women in science form more diverse research networks than men? An analysis of Spanish biomedical scientists
Source: PLoS One. 2020 Aug 27;15(8):e0238229. doi: 10.1371/journal.pone.0238229 (PMC7451541; doi:10.1371/journal.pone.0238229)
Supplement: S6 Table — (DOCX) [file pone.0238229.s006.docx]

**S6 Table. Results for the regressions including the interplay between gender and PI.**

|  | **Model 1 Partner diversity** | | | **Model 2 Openness** | | | **Model 3 Range of brokerage roles** | | | **Model 4 Consultant** | | | **Model 5 Liaison** | | |
| --- | --- | --- | --- | --- | --- | --- | --- | --- | --- | --- | --- | --- | --- | --- | --- |
|  | ***β*** | **S.E.** | **p** | ***β*** | **S.E.** | **p** | ***β*** | **S.E.** | **p** | ***β*** | **S.E.** | **p** | ***β*** | **S.E.** | **p** |
| *Explanatory variables* |  |  |  |  |  |  |  |  |  |  |  |  |  |  |  |
| Woman | 0.107 | 0.056 | 0.057 | 0.013 | 0.027 | 0.633 | 0.010 | 0.013 | 0.417 | 0.213 | 0.246 | 0.386 | 0.459 | 0.275 | 0.095 |
| Principal Investigator | **0.145** | 0.069 | **0.035** | 0.058 | 0.032 | 0.065 | 0.015 | 0.016 | 0.356 | -0.424 | 0.242 | 0.081 | 0.078 | 0.317 | 0.806 |
| Woman*Principal Investigator | -0.048 | 0.076 | 0.529 | -0.016 | 0.038 | 0.685 | 0.015 | 0.021 | 0.451 | 0.348 | 0.308 | 0.259 | 0.042 | 0.366 | 0.908 |
| *Control variables* |  |  |  |  |  |  |  |  |  |  |  |  |  |  |  |
| *Individual level* |  |  |  |  |  |  |  |  |  |  |  |  |  |  |  |
| Breadth of skills | -0.001 | 0.012 | 0.906 | -0.008 | 0.006 | 0.177 | -0.002 | 0.003 | 0.424 | **-0.140** | 0.041 | **0.001** | -0.069 | 0.056 | 0.220 |
| Tertius iungens | **0.074** | 0.021 | **0.000** | -0.006 | 0.011 | 0.600 | **0.010** | 0.005 | **0.034** | **0.322** | 0.085 | **0.000** | 0.158 | 0.123 | 0.201 |
| Age | 0.000 | 0.002 | 0.931 | -0.001 | 0.001 | 0.568 | **0.001** | 0.170 | **-0.001** | 0.000 | 0.008 | 0.963 | 0.005 | 0.013 | 0.705 |
| Conscientiousness | -0.001 | 0.021 | 0.955 | -0.003 | 0.010 | 0.769 | -0.004 | 0.005 | 0.386 | 0.118 | 0.076 | 0.120 | 0.044 | 0.095 | 0.646 |
| Neuroticism | -0.008 | 0.018 | 0.635 | 0.003 | 0.009 | 0.776 | 0.002 | 0.004 | 0.531 | -0.101 | 0.067 | 0.131 | -0.091 | 0.101 | 0.369 |
| Openness (personality) | 0.005 | 0.025 | 0.844 | 0.020 | 0.012 | 0.096 | 0.010 | 0.006 | 0.100 | **0.207** | 0.099 | **0.037** | 0.057 | 0.119 | 0.631 |
| Extraversion | 0.011 | 0.017 | 0.501 | 0.013 | 0.009 | 0.165 | 0.000 | 0.005 | 0.949 | -0.020 | 0.066 | 0.758 | 0.031 | 0.103 | 0.764 |
| Agreeableness | 0.013 | 0.024 | 0.579 | 0.005 | 0.013 | 0.690 | 0.004 | 0.006 | 0.491 | 0.134 | 0.085 | 0.114 | -0.076 | 0.125 | 0.541 |
| Intrinsic motivation | -0.032 | 0.027 | 0.236 | **-0.030** | 0.014 | **0.030** | **-0.014** | 0.007 | **0.030** | -0.216 | 0.100 | 0.032 | -0.108 | 0.139 | 0.437 |
| Extrinsic motivation | 0.016 | 0.018 | 0.388 | -0.005 | 0.009 | 0.567 | -0.002 | 0.004 | 0.682 | -0.004 | 0.070 | 0.953 | -0.108 | 0.093 | 0.244 |
| Basic orientation | **-0.115** | 0.040 | **0.004** | 0.034 | 0.022 | 0.120 | -0.001 | 0.010 | 0.932 | 0.024 | 0.138 | 0.865 | **-0.701** | 0.200 | **0.000** |
| Network size | **0.107** | 0.008 | **0.000** | **0.034** | 0.004 | **0.000** | **0.076** | 0.002 | **0.000** | **0.588** | 0.033 | **0.000** | **0.584** | 0.043 | **0.000** |
| Creative self-efficacy | 0.000 | 0.027 | 0.993 | -0.009 | 0.015 | 0.535 | 0.001 | 0.006 | 0.868 | -0.095 | 0.103 | 0.358 | 0.185 | 0.134 | 0.168 |
| MNCS | -0.004 | 0.017 | 0.823 | 0.003 | 0.007 | 0.634 | 0.002 | 0.003 | 0.437 | 0.030 | 0.032 | 0.345 | -0.046 | 0.083 | 0.578 |
| *Research Group* |  |  |  |  |  |  |  |  |  |  |  |  |  |  |  |
| Group network density | -0.028 | 0.082 | 0.736 | **-0.079** | 0.040 | **0.049** | **-0.049** | 0.019 | **0.010** | **-0.564** | 0.261 | **0.031** | -0.574 | 0.388 | 0.139 |
| Group network frequency | 0.002 | 0.027 | 0.936 | 0.020 | 0.015 | 0.179 | 0.001 | 0.007 | 0.902 | -0.167 | 0.111 | 0.132 | -0.114 | 0.171 | 0.505 |
| Share of females per group | **-0.003** | 0.001 | **0.044** | 0.000 | 0.001 | 0.692 | 0.000 | 0.000 | 0.639 | 0.004 | 0.005 | 0.421 | -0.008 | 0.007 | 0.217 |
| Team size | 0.000 | 0.002 | 0.909 | -0.001 | 0.001 | 0.377 | 0.000 | 0.000 | 0.302 | 0.007 | 0.008 | 0.382 | 0.012 | 0.009 | 0.154 |
| *Organizational level* |  |  |  |  |  |  |  |  |  |  |  |  |  |  |  |
| CIBER dummies |  | Yes |  |  | Yes |  |  | Yes |  |  | Yes |  |  |  |  |
| University | 0.031 | 0.049 | 0.527 | 0.044 | 0.026 | 0.090 | **0.037** | 0.012 | **0.003** | 0.316 | 0.203 | 0.120 | 0.187 | 0.251 | 0.456 |
| Hospital | 0.053 | 0.050 | 0.289 | -0.005 | 0.027 | 0.859 | 0.016 | 0.013 | 0.237 | **0.596** | 0.202 | **0.003** | 0.355 | 0.254 | 0.163 |
| Constant | -0.237 | 0.314 | 0.450 | 0.076 | 0.177 | 0.668 | -0.094 | 0.080 | 0.241 | **-4.367** | 1.114 | **0.000** | -1.453 | 1.516 | 0.338 |
| Cox & Snell pseudo R2 |  | 0.285 |  |  | 0.127 |  |  | 0.693 |  |  | 0.281 |  |  | 0.157 |  |
| N |  | 897 |  |  | 897 |  |  | 897 |  |  | 897 |  |  | 897 |  |

Note: To investigate Model 1, Model 2 and Model 3 a Tobit regression is used, while a Negative Binomial model is employed for Model 4 and Model 5.
