# Supplementary material for: Do women in science form more diverse research networks than men? An analysis of Spanish biomedical scientists
Source: PLoS One. 2020 Aug 27;15(8):e0238229. doi: 10.1371/journal.pone.0238229 (PMC7451541; doi:10.1371/journal.pone.0238229)
Supplement: S3 File — (DOCX) [file pone.0238229.s009.docx]

**S3. File. QQ-plots for partner diversity (DV1), openness (DV2) and range of brokerage roles (DV3).**
